# *Leishmania infantum* and *Leishmania braziliensis:* Differences and Similarities to Evade the Innate Immune System

**DOI:** 10.3389/fimmu.2016.00287

**Published:** 2016-08-03

**Authors:** Sarah de Athayde Couto Falcão, Tatiana M. G. Jaramillo, Luciana G. Ferreira, Daniela M. Bernardes, Jaime M. Santana, Cecília B. F. Favali

**Affiliations:** ^1^Department of Cell Biology, Biology Institute, University of Brasília, Brasília, Brazil; ^2^Núcleo de Medicina Tropical (NMT), University of Brasília, Brasília, Brazil

**Keywords:** apoptosis, dendritic cells, *Leishmania infantum*, *Leishmania braziliensis*, CD209

## Abstract

Visceral leishmaniasis is a severe form of the disease, caused by *Leishmania infantum* in the New World. Patients present an anergic immune response that favors parasite establishment and spreading through tissues like bone marrow and liver. On the other hand, *Leishmania braziliensis* causes localized cutaneous lesions, which can be self-healing in some individuals. Interactions between host and parasite are essential to understand disease pathogenesis and progression. In this context, dendritic cells (DCs) act as essential bridges that connect innate and adaptive immune responses. In this way, the aim of this study was to compare the effects of these two *Leishmania* species, in some aspects of human DCs’ biology for better understanding of the evasion mechanisms of *Leishmania* from host innate immune response. To do so, DCs were obtained from monocytes from whole peripheral blood of healthy volunteer donors and from those infected with *L. infantum* or *L. braziliensis* for 24 h. We observed similar rates of infection (around 40%) as well as parasite burden for both *Leishmania* species. Concerning surface molecules, we observed that both parasites induced CD86 expression when DCs were infected for 24 h. On the other hand, we detected a lower surface expression of CD209 in the presence of both *L. braziliensis* and *L. infantum*, but only the last one promoted the survival of DCs after 24 h. Therefore, DCs infected by both *Leishmania* species showed a higher expression of CD86 and a decrease of CD209 expression, suggesting that both enter DCs through CD209 molecule. However, only *L. infantum* had the ability to inhibit DC apoptotic death, as an evasion mechanism that enables its spreading to organs like bone marrow and liver. Lastly, *L. braziliensis* was more silent parasite, once it did not inhibit DC apoptosis in our *in vitro* model.

## Introduction

Leishmaniasis is a vector-borne disease that commits millions of people around the world. In 2013, around 215,000 new cases were reported in the world. Of these, around 21,000 were reported in Brazil (1). Around 0.2–0.4 and 0.7–1.2 million visceral leishmaniasis (VL) and cutaneous leishmaniasis (CL), respectively, occur each year. Over 90% of new cases of VL occur in six countries all over the world, including Brazil. About 95% of CL cases occur in the Americas, the Mediterranean basin, and the Middle East and Central Asia (2).

In the sandfly, the flagellated motile forms of *Leishmania* spp. called promastigotes progress through several morphological stages of differentiation, regulating the vector midgut environment (3). Finally, it becomes the non-dividing, infectious metacyclic promastigotes that are transmitted during a sandfly bite, when they are able to infect or be phagocyted by professional phagocytes as macrophages (4) and dendritic cells (DCs) (5). The parasite inside host cells becomes amastigote, a stage without an externalized flagellum that is capable of multiplication in antigen-presenting cells (6). CL is the most common clinical form of leishmaniasis and causes localized skin lesions, especially in arms and legs. In the New World, *Leishmania braziliensis* is able to cause from localized self-limited lesions to tissue destructive mucosal forms (7) that can worsen with age (8). On the other hand, visceral forms are caused by *Leishmania infantum* (9), and the disease, characterized by fever, weight loss, enlargement of the spleen and liver, and anemia, is fatal if left untreated (10).

Host–parasite interactions during innate immune responses determine the fate of adaptive immunity, contributing to healing or parasite persistence in leishmaniasis (11). DCs are professional antigen-presenting cells that interact with pathogens in peripheral tissues and stimulate T lymphocytes after migration to secondary lymphoid organs (12). In the periphery, DCs are in an immature state, with high potential to perform phagocytosis through many receptors that recognize pathogen-associated molecular patterns, as DC-SIGN (CD209) (13–15). Toll-like receptors (TLRs) are also involved in innate response to *Leishmania* parasites. During murine *Leishmania major* infection, data in literature showed that TLR9 was required for the induction of IL-12 in bone marrow-derived DCs (BMDCs) by intact *L. major* parasites or *L. major* DNA. This IL-12 production was essential for early interferon-gamma expression and NK cell activation (16). After pathogen internalization, DC is able to migrate from periphery to secondary tissues, rising MHC expression as well as other co-stimulatory molecules CD86 and CD83. After all these events, DCs are able to properly perform antigen presentation to T cells and determinate the fate of adaptive immune response (17). DCs can also become tolerogenic cells, as observed in the physiologic regulation of apoptosis (18). When *L. braziliensis* were in contact with murine CD11c^+^ DCs, they were able to produce high levels of IL-12p70 as well as stimulate a significant expression of CD40 and CD83 in the surface of these DCs (19). Another study from the literature, working with mice BMDCs, showed that *L. infantum* could infect and survive inside these cells. Besides, they observed that *L. infantum* promastigotes were not able to upregulate CD40 and CD86 surface expression. The authors also observed that *L. infantum* was able to induce some level of IL-12p40 and IL-10, with no differences in TNF-α levels (20). Another group showed that bystander BMDCs from Balb/c mice increased IL-12p40 and expressed more CD40, CD86, and MHC class II in the cell surface than infected or not exposed cells. These bystander DCs induced a protective CD4^+^ IFN-γ T cells response, while *L. infantum*-infected DCs polarizing to T-bet+IFN-γ+IL-10+ double producer T cells phenotype (21). On the other hand, human DCs infected with *L. major* showed an increase of HLA-DR, CD86, and CD40. When *L. major*-infected DCs were cocultured with T lymphocytes and treated with anti-CD40 ligand (CD40L), IL-12p70 and IFN-γ production decreased, concluding that IL-12p70 and IFN-γ production are CD40L-dependent (22). When CD86 (B7-2)^−/−^ mice were infected with *L. major*, they presented a resistance, differently CD80 (B7-1)^−/−^, and wild-type Balb/c mice maintained its susceptibility. Moreover, CD86^−/−^ produced more IL-4 than wild-type mice, suggesting that CD86 induced a Th2 response (23). Infection of BALB/c mice by *Leishmania amazonensis* led to higher accumulation of Langerhans cells, and CD4^+^ and CD8^+^ T cells were found that produced IL-4 and IL-10. Nevertheless, *L. braziliensis* infection induced dermal DCs accumulation, increased effectors, and activated memory CD4^+^ T cells and IL-4, IL-10, IFN-γ production by CD4^+^ and CD8^+^ T cells (24).

Many pathogens alter DC biology, favoring its persistence in the infected host. Data in literature showed that *L. amazonensis* is able to inhibit human DC differentiation from monocytes (25) and several intracellular signaling pathways (26), altering DC biology and function. Data from murine models of leishmaniasis show that inhibitory pathways are also involved in disease pathogenesis. One example is the OX40L–OX40 pathway that enhances Th2 responses in *L. major*-infected mice (27). Working with *Ox40l*^−/−^ mice, the authors observed that they were very susceptible to *L. major* and *Leishmania mexicana*. Interestingly, only lymphnode cells from *L. major Ox40l*^−/−^ mice produced less IL-4 and IL-10 than *Ox40l*^+/+^. In this way, this pathway is relevant to Th2 development only in the context of *L. major* infection, but it does not alter *L. major* outcome of infection (28, 29). Other surface receptors, the programmed death ligand 1 (PD-L1) and 2 (PD-L2), were studied in susceptible mice infected with *L. mexicana*. PD-L1 knockout mouse demonstrated resistance, decrease of lesion size, as well as parasitic load and IL-4 production after infection. Although PD-L2 knockout mouse showed increase of lesion size, parasitic load, and antibodies production, no difference on IFN-γ and IL-4 production was observed. Both receptors play different roles in response to *L. mexicana* (30).

Another way that pathogens have to adapt to the host is to alter cell death. It is well known that *Mycobacterium tuberculosis* induces cell death through apoptosis, but this event favors antigen presentation (31). Moreover, pathogens as *Leishmania* delay cell death as a way to survive inside host cell. For example, apoptosis of monocyte-derived dendritic cells (moDCs) induced by treatment with camptothecin was downregulated by infection with *L. mexicana* amastigotes, detected by annexin V binding to phosphatidylserine (32).

It is not clear how the regulation of human DC biology is affected in different ways by species of *Leishmania*. In this way, the aim of this study is to analyze the expression of surface molecules relevant to antigen processing and presentation as well as DCs survival after interaction with *L. infantum* or *L. braziliensis*.

## Materials and Methods

### Parasites

*Leishmania infantum* (MHOM/BR/1974/PP75) and *L. braziliensis* (MHOM/BR/01/BA788) promastigotes were maintained in Schneider’s medium (SIGMA) with 20% of calf fetal serum (GIBCO) and gentamicin (50 μm/mL). *Leishmania* stationary-phase promastigotes were quantified in Neubauer chamber. After counting, promastigotes were harvested from culture bottles, washed three times with cold PBS (800 g for 10 min), and adjusted in RPMI medium to be added in DC culture.

### Dendritic Cell Culture

Human DCs were differentiated from monocytes. Briefly, venous blood was collected from volunteer healthy donors (*n* = 8), and peripheral blood mononuclear cells (PBMC) were obtained by passage over a Ficoll gradient (GE Healthcare). Cells were harvested, washed, and stained with antibodies, anti-CD14, conjugated with microbeads (Miltenyi Biotec). Stained cells (monocytes) were purified after positive selection in a magnetic field. Monocytes were then counted and cultivated in RPMI 1640 medium supplemented with 2 mM l-glutamine, 100 U/mL penicillin, 100 mg/mL streptomycin (GIBCO), IL-4 (800 IU/mL), and GM-CSF (50 μg/mL) (both from PeproTech) with 10% of fetal serum bovine (GIBCO) in 24-wells plates (5 × 10^5^ cells/wells) for 7 days at 37°C and 5% CO_2_. New medium (200 μL) supplemented with cytokines was added on days 3 and 6 of the culture. On day 7, DCs were infected (1 DC:10 parasites), cultivated in 24-wells plates for 24 h at 37°C and 5% CO_2_, harvested, and characterized by flow cytometry (FACS Verse BD Biosciences). All experimental conditions and measurements were performed with the same donors.

### Flow Cytometry and Antibodies

Dendritic cells were cocultured with *Leishmania* species for 24 h, and control cells were harvested and washed with PBS. Cells were then resuspended in FACS buffer (5 × 10^6^ cells/mL) and incubated at 4°C for 20 min. The cells were stained with CD1a FITC-conjugated (clone HI149), HLA-DR PE-conjugated (clone LN3), CD86 PE-Cy5-conjugated (clone IT2-2), and CD209 PE-conjugated (clone eB-h209) (all eBioscience) and incubated for 30 min at 4°C. Finally, cells were washed, resuspended in FACS buffer, and acquired on a flow cytometer. For cell death experiments, after infection time, the cells were washed and resuspended in 195 μL of binding buffer to each 2–5 × 10^5^ cells, added 5 μL of annexin V, and incubated for 10 min at room temperature. The cells were washed with PBS 1×, resuspended in 190 μL of binding buffer, added 10 μL propidium iodide (PI) (annexin V and propidium iodide kit, eBioscience), and acquired on a flow cytometer. Stained cells were acquired on a Verse flow cytometer (BD Bioscience) and analyzed using FlowJo Software (Tree Star, Inc.).

### Cytokine Assay

Dendritic cells were infected with both *Leishmania* species. After 24 h of infection, supernatant was collected and level of TNF-α was measured by ELISA. TNF-α was quantified (test detection range from 20 to 1000 pg/mL) with purified antihuman TNF-α, antihuman TNF-α biotin, and streptavidin–alkaline phosphatase, according to the instruction of suppliers (Novex, Life Technologies, Invitrogen). Absorbance was measured and analyzed with the program SoftMax Pro (Molecular Devices).

### Ethical Statement

Human blood samples were collected after the signature of an informed consent signed by all volunteers, and the project was approved by The Ethical Committee for Human Beings from the Medicine Faculty of University of Brasilia (approval no. 072/2009). Samples were collected by venous puncture by a trained and specialized laboratory technician in the Universidade de Brasília.

### Statistical Analysis

The statistical analysis was conducted using GraphPad Prism Software. Samples were tested by Shapiro–Wilk normality test, and the significance of the results was calculated using parametrical paired *t* test, and a *p*-value of <0.05 was considered significant.

## Results

### *L. braziliensis*- or *L. infantum*-Exposed Dendritic Cells Upregulate CD86 and Downregulate CD209 Expression

**Figure 1:**
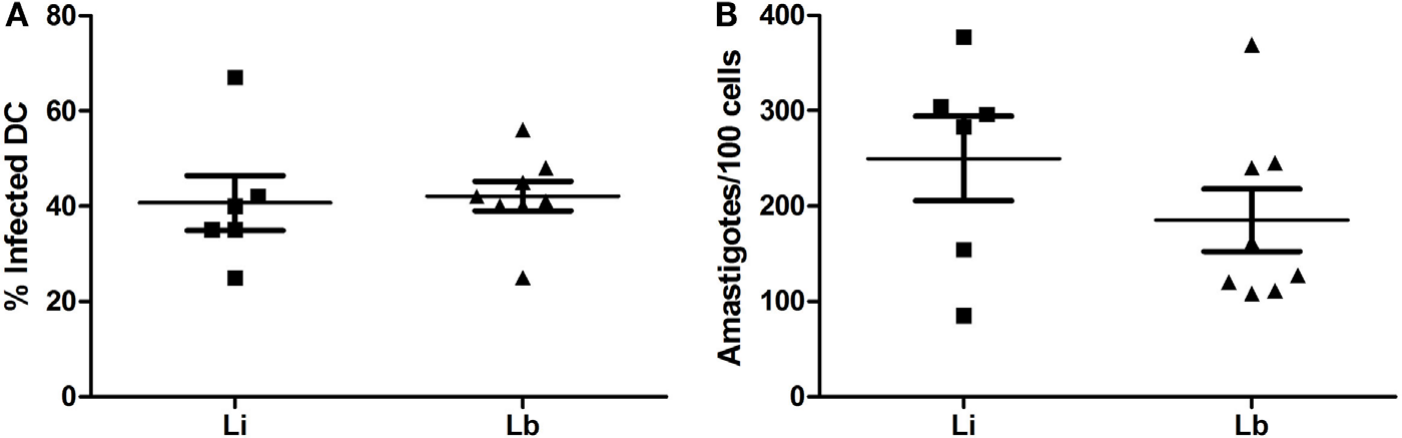
**Dendritic cells’ infection by *L. infantum* (Li) or *L. braziliensis* (Lb) – immature DCs were infected in a rate of 10 *Leishmania* to 1 DC**. Infection after 24 h, in hematoxylin–eosin (HE)-stained slides. Median ± SD. Each point represents one donor. *p < 0.01. **(A)** Percentage of infected DCs. **(B)** Amastigotes per 100 cells.

**Figure 2:**
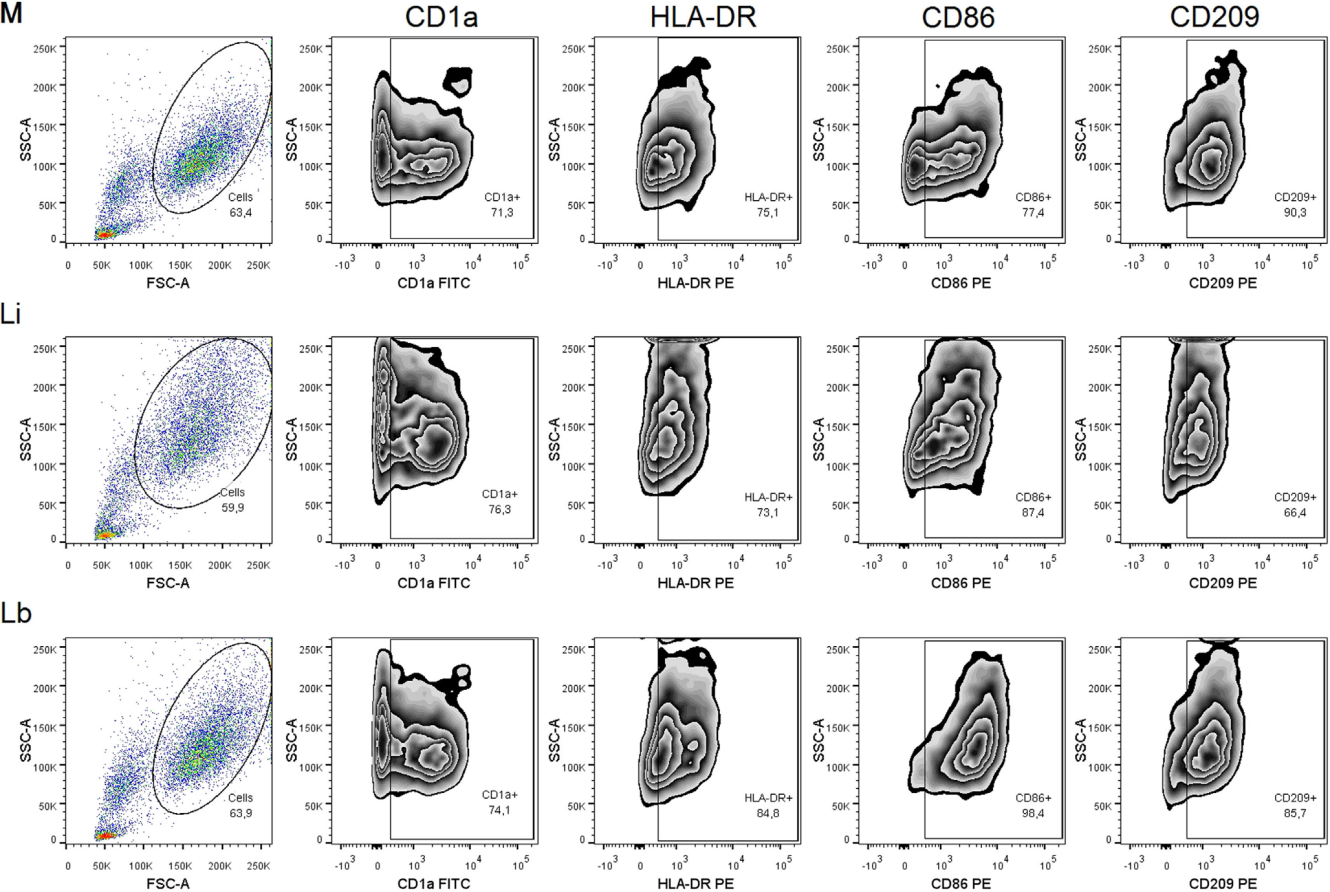
**Gating strategy to analyze DCs’ regulation by *Leishmania* parasites – DCs in control and infected conditions were gated by granularity and size and further analyzed for expression of CD1a/HLA-DR/CD86/CD209 surface molecules**. M, medium, Li, *Leishmania infantum*, Lb, *Leishmania braziliensis*.

**Figure 3:**
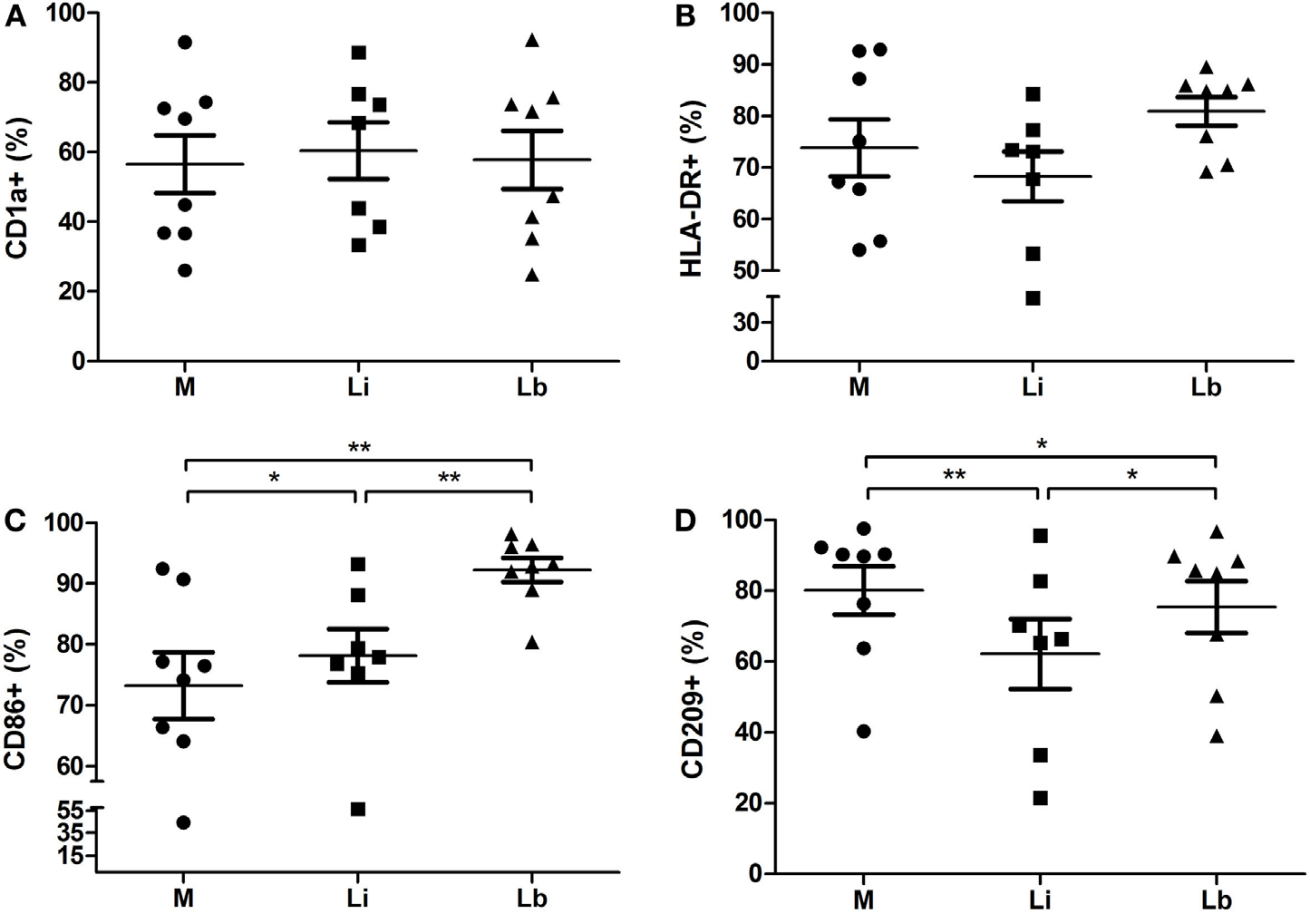
***L. braziliensis*- or *L. infantum-*exposed dendritic cells upregulate CD86 and downregulate CD209 expression – flow cytometry analysis of surface molecule expression in immature DCs differentiated from human monocytes with cytokine-conditioned media (M) or conditioned media in the presence of viable *L. infantum* (Li) or *L. braziliensis* (Lb)**. CD1a **(A)**, HLA-DR **(B)**, CD86 **(C)**, and CD209 **(D)**. Median ± SD. Each point represents one donor. *p < 0.05, **p < 0.01.

After 24 h, the parasite load of DCs was accessed by optic microscopy, and we observed 40.67 ± 5.8% *L. infantum* (Li)-infected DCs and 42.13 ± 3.1% *L. braziliensis* (Lb)-infected DCs (Figure 1A). The amastigotes number per 100 cells was 249.8 ± 44.23 amastigotes in *L. infantum* (Li)-infected DCs and 185.3 ± 32.73 in *L. braziliensis* (Lb)-infected cells (Figure 1B). Later, we decided to observe the co-stimulatory and surface molecules expression by DCs after the exposure to *L. infantum* or *L. braziliensis*. Then, we stained the cultured DCs with medium or each parasites species with anti-CD1a, anti-HLA-DR, anti-CD86, and anti-CD209 antibodies. The cells were gated by granularity and size, and the gated cells were further analyzed for expression of CD1a/HLA-DR/CD86/CD209 surface molecules (Figure 2). Control DCs, differentiated with media only (M), showed 56.53 ± 8.2% of CD1a expression. This was very similar in DCs exposed to *L. infantum* (60.40 ± 8.1%) as well as exposed to *L. braziliensi*s (57.8 ± 8.3%) (Figure 3A).

In the same way, we observed a consistent higher expression of HLA-DR in control cells (73.81 ± 5.55%)-, in *L. infantum* (68.27 ± 4.8%)-, and *L. braziliensis* (80.89 ± 2.7%)-exposed DCs, with no significant differences between the groups (Figure 3B).

However, both *Leishmania* were able to induce a significant raise in CD86 expression. We observed that CD86 expression was 73.23 ± 5.4% in control cells and increased to 78.16 ± 4.3% in the presence of *L. infantum* and to 92.24 ± 1.9% in the presence of *L. braziliensis* (Figure 3C). Moreover, this increase was significantly higher in *L. braziliensis*-infected DCs than in *L. infantum*-infected DCs (Figure 3C).

On the other hand, we observed that both *Leishmania* were able to inhibit significantly CD209 expression. Its expression was 80.09 ± 6.8% in control cells, while in the presence of *L. infantum*, it was down modulated to 62.17 ± 9.88%. The presence of *L. braziliensis* also led to a lower CD209 expression (75.36 ± 7.3%) (Figure 3D). This decrease was significantly lower in *L. infantum*-infected DCs than *L. braziliensis*-infected DCs (Figure 3D).

**Figure 4:**
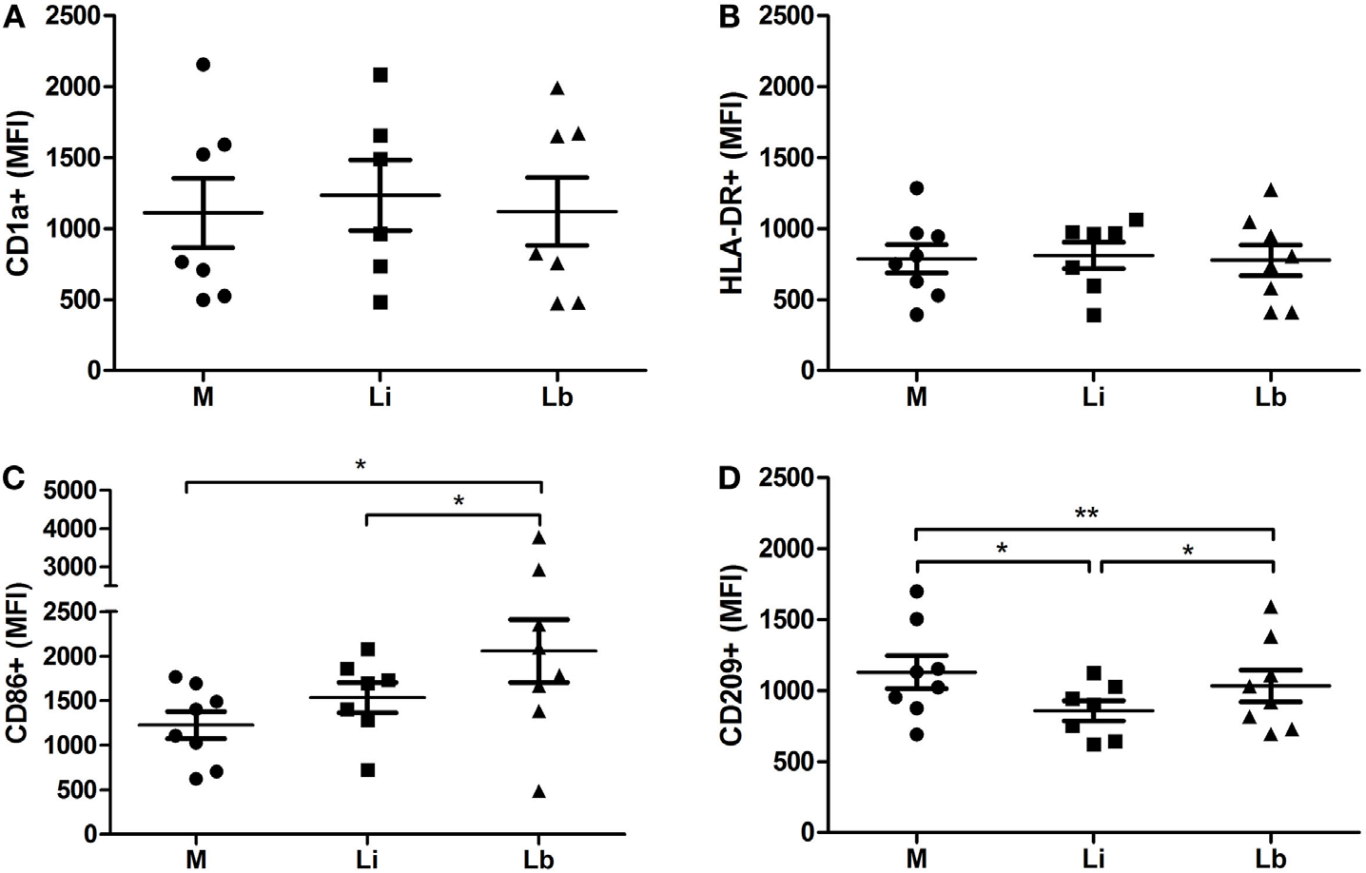
***L. braziliensis-*exposed DCs increase the mean of fluorescence intensity (MFI) of CD86 and decrease CD209 – flow cytometry analyzes of the MFI in immature DCs differentiated from human monocytes with cytokine-conditioned media (M) or conditioned media in the presence of viable *L. infantum* (Li) *or L. braziliensis* (Lb)**. CD1a **(A)**, HLA-DR **(B)**, CD86 **(C)**, and CD209 **(D)**. Median ± SD. Each point represents one donor. *p < 0.05, **p < 0.01.

When we analyzed the mean intensity of fluorescence (MFI) of these molecules, we observed the same effects that we observed in the percentage of expression. The MFI was similar in all experimental conditions for CD1a and HLA-DR (Figures 4A,B). On the other hand, we observed a significantly higher CD86 MFI induced by *L. braziliensis* (2060 ± 351.9), but no significant difference was found in the presence of *L. infantum* (1537 ± 169.4) when compared to control cells (1227 ± 152.2) (Figure 4C). Also, as we observed in surface expression, the CD209 MFI was significantly lower in the presence of *L. infantum* (856.1 ± 72.8) or *L. braziliensis* (1033 ± 112.6), compared to control cells (1127 ± 116.7) (Figure 4D).

### Exposure to *L. infantum* but Not *L. braziliensis* Decreases the Dendritic Cells’ Cellular Death

**Figure 5:**
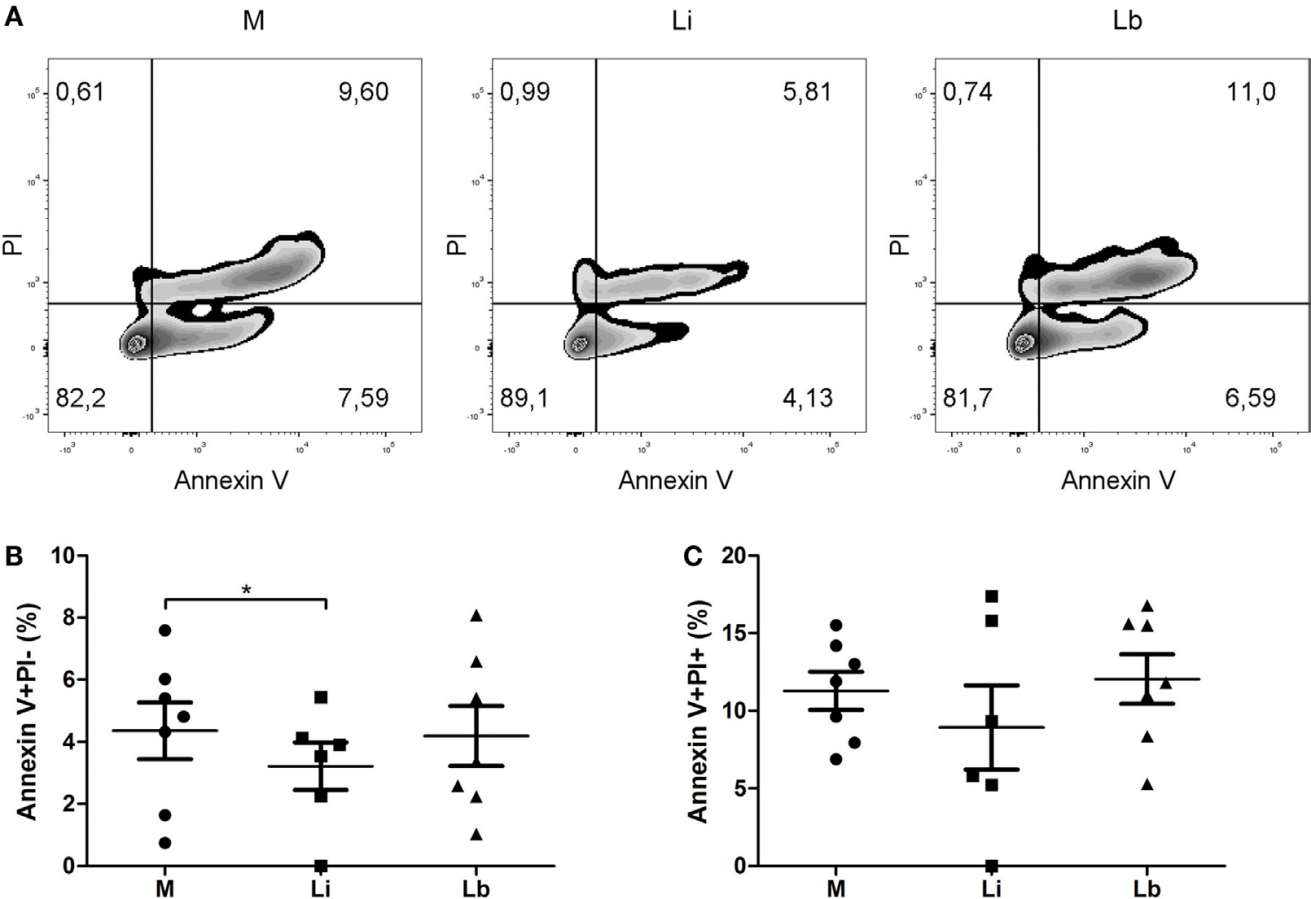
***L. infantum* delayed the cellular death of dendritic cells – flow cytometry gating strategy (A) to perform analyses of annexin V and propidium iodide (PI) staining of control DCs (M) or treated with viable *L. infantum* (Li) or *L. braziliensis* (Lb)**. Annexin V positive cells **(B)** and annexin V and PI positive cells **(C)**. Median ± SD. Each point represents one donor. *p < 0.05, **p < 0.01.

Next, we decided to evaluate if these two *Leishmania* species were able to induce or not cell death of DCs. To do so, after a coculture of the DCs with the two parasite species, we stained the cells with annexin V and PI (Figure 5A). We observed no significant differences between DCs only and *L. braziliensis*-exposed DCs in the annexin V+PI− and annexin V+PI+ populations (Figures 5B,C, respectively). Nevertheless, the cell population that was annexin V+PI− population significantly decreased in *L. infantum*-exposed DCs, from 5.7 ± 1.0% in control cells to 2.9 ± 0.6%, that is a 50.8% decrease in DC death, showing that the *L. infantum* is able to inhibit cellular death of DCs (Figure 5B). However, no significant decrease was observed in the annexin V+PI+ population, 11.36 ± 2% in control cells and 8.3 ± 2.4% in *L. infantum*-exposed DCs (Figure 5C).

### *L. infantum*- and *L. braziliensis*-Exposed Dendritic Cells Produced TNF-α

**Figure 6:**
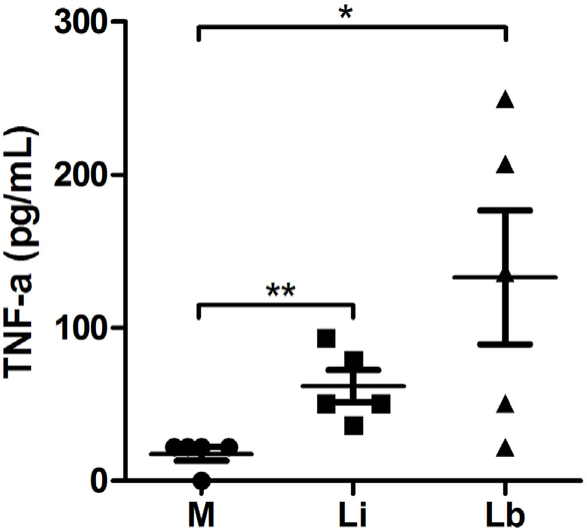
***L. braziliensis* induced TNF-α secretion on immature DCs – cytokine levels measured on culture supernatants after 24 h of infection**. Control DCs were cultured in cytokine-conditioned media only or treated with viable *L. infantum* or *L. braziliensis* parasites. Median ± SD. Each point represents one donor. *p < 0.05, **p < 0.01.

Coculture with *L. infantum* or *L. braziliensis* triggered a significant secretion of TNF-α by DCs. Control cells produced 17.6 ± 4.4 pg/mL, while DCs cocultured with *L. infantum* induced 61.9 ± 10.4 pg/mL, a 3.5-fold significant increase in this cytokine secretion. On the other hand, *L. braziliensis* stimulated significantly even more TNF-α secretion (133.2 ± 43.7 pg/mL), almost an eightfold increase (Figure 6).

## Discussion

Many data in literature show the relevant role of DCs in diverse pathogen–host interactions, especially in the initial events of innate immunity. These initial events lead the course of adaptive response (33). For a better understanding of *Leishmania* and infected host regulation, we initially cocultured DCs with stationary non-opsonized *L. braziliensis* and *L. infantum* promastigotes for 24 h. The infection rate was similar in our experiments between the two *Leishmania* species (around 40%), and we found no significant difference of internalized parasites number. Concerning infection data, non-opsonized *L. mexicana* promastigotes presented higher phagocytosis than opsonized promastigotes or amastigotes by human moDCs after 3 h. Six hours later, opsonized promastigotes showed higher phagocytosis than non-opsonized promastigotes and amastigotes, but no difference was observed after 24 h of infection (5). Infection of human DCs with *L. amazonensis* showed a similar rate of infection, around 45%, and a parasite burden median of six amastigotes per DC (25).

We observed that both *L. braziliensis* and *L. infantum* were able to induce an increase in CD86 expression and also caused a decrease in CD209 (DC-SIGN) expression in human DCs. A study with *L. amazonensis* showed that the parasite modulates the co-stimulatory molecules expression during the differentiation of human moDCs, affecting the mixed leukocyte reaction and the specific T lymphocytes responses. Similar to our data, *L. amazonensis* also caused an increase in CD86 expression after a 24-h parasite DCs contact (25). On the other hand, murine mature DCs infected with *L. amazonensis* amastigotes presented a decreased antigen presentation, CD40 and CD83 expression, and also a reduced CD4^+^ T cells priming (34). Contreras et al. (35), showed that *L. mexicana* infection also interfered in the DC maturation process, decreasing co-stimulatory molecules expression and antigen presentation, injuring the presentation to T cells by human DCs. Macrophages cultured with peripheral blood leukocytes (PBL) exhibited CD40 and CD86 expression in the absence of *L. major*, but their expression levels were higher in the presence of the parasite. After blocking CD80, CD86, both, or CD40, macrophages cultured with PBL and *L. major* produced less IFN-γ, IL-5, and IL-12, showing that these co-stimulatory pathways were important to the development of an anti-*Leishmania*-efficient immune response (36). On the other hand, a study performed with VL patients showed that the subset of inflammatory monocytes CD14^+^CD16^+^ remained the same as in the control group. Similarly, the population of CD54^+^HLA-DR^+^ monocytes was significantly downregulated during active disease. Finally, they observed an increase in CD80 expression level, but a decreased CD86 expression (37). Concerning infection with *L. braziliensis*, data from literature showed that CD14^+^ monocytes of peripheral blood from CL patients had a decreased expression of CD80 and CD86 following culture with media only or after restimulation with *Leishmania* antigen (38). It seems that monocytes and DCs have different roles in the regulation leishmaniasis. Monocytes seem to have a role in regulating the lesion site during CL pathogenesis, and the authors mentioned before Vieira et al. (38) observed a diminished expression of B7 molecules. In our model, we worked with moDCs from healthy donors’ peripheral blood. In this way, it is possible that in these initial interactions, once our experiments were performed after a 24-h infection with *Leishmania* parasites, these DCs regulate CD86 expression positively, being able to activate naive lymphocytes. On the other hand, the effects seem to be opposite for monocytes that migrate to lesion sites, once in patients with CL it is observed a negative regulation of CD86 (38).

Concerning CD209 expression, the decrease of this surface molecule was also observed in *L. major*- and *L. donovani*-infected human DCs (39). It is well established that the receptor CD209 can be used for internalizing microrganisms as parasites (40). In this way, our data are in accordance with data in the literature, suggesting that *L. infantum* and *L. braziliensis* attach to CD209 to access host DCs, which would explain the decrease in CD209 in the surface, as it could be internalized. Nevertheless, CD209 was involved in the phagocytosis of *L. mexicana* promastigotes after 3 h, but not after 6 or 24 h (5). Excreted or secreted recombinant proteins of *L. infantum* interfered in the differentiation of DCs, decreasing the HLA-DR expression but increasing the CD86 expression (41). We observed the same effect concerning CD86, but we observed no significant differences in HLA-DR surface expression caused by *L. infantum* or *L. braziliensis* in our work. Regarding CD86 and CD209 expression, we found a higher expression of CD86 in the presence of *L. braziliensis* than in the presence of *L. infantum*; however, the decrease in CD209 expression was greater in cells infected with *L. infantum* than in *L. braziliensis*-infected ones. Though, the rates of infection as well as the number of internalized amastigotes were similar between both the species of *Leishmania*. This can be explained by the fact that DCs have other recognition receptors that are able to recognize and be involved in *Leishmania* internalization, which were not investigated in this work. We can mention the mannose–fucose receptor, which is present in DCs that can recognize lipophosphoglycan (LPG), GP63, and proteophosphoglicans (PPG) present in the membrane of *Leishmania* species (42, 43). This pathway could explain the DCs’ infection rate by *L. braziliensis* and the number of amastigotes to be similar to the *L. infantum*-infection. However, macrophages of B6 mice mannose receptor (MR) knockout showed an increase of parasite load after infection with *L. infantum*. This group showed that MR and Dectin-1 signaling pathways are essential to trigger the oxidative burst in macrophages infected with *L. infantum*, enabling parasite control (44).

Many data in literature showed the relevance of cytokines regulation in leishmaniasis pathogenesis. The presence of *L. amazonensis* during the differentiation of human DC inhibited IL-6 and IL-10 secretion. Besides, these DCs cocultured with *L. amazonensis*, when in contact with autologous lymphocytes, caused a lower secretion of IFN-γ (25). Moreover, amastigotes of *L. amazonensis* suppressed IL-12+p40 and IL-10 production by murine DCs (34). However, we found a higher release of TNF-α after coculture of DCs and *L. braziliensis* or *L. infantum*. The excreted or secreted recombinant proteins of *L. infantum* also induced TNF-α production (41), as we saw for both *Leishmania* species in this work. Studies with fluorescent *L. braziliensis* parasites allowed the observation that the TNF-α production derived from infected DCs, but not from bystander DCs, had contact with *L. braziliensis* in the medium but were not infected. Moreover, soluble parasite products and the TNF-α released by infected DCs provided a more efficient antigen presentation by DCs (45). Data from literature evaluated TNF-α production from PBMC of patients and healthy individuals after stimulation with soluble *L. braziliensis* antigens. The results showed a range in the SD, as expected for human individuals. They showed that healthy donors produced 48.6 ± 77.18 pg/mL of TNF-α (46). Another study that compared PBMC responses from patients with CL and mucosal leishmaniasis (ML), after stimulation with *L. braziliensis* soluble antigen, also detected a wide range of TNF-α levels. In CL, TNF-α levels ranged from 0 to 7440 pg/mL, while in ML patients from 892 to 9173 pg/mL (47). These differences in TNF levels are expected and may exist due to the genetic constitution of the host. On the other hand, data from literature that analyzed immune responses from patients with active VL detected some similarities among VL patients and healthy individuals, concerning cytokines regulation. Analysis *ex vivo* of patient’s cells with active VL showed the production of high levels of anti-inflammatory cytokines and lower TNF levels, the last ones very similar to healthy controls (37). Another study with patients with active VL due to *L. infantum* also showed lower TNF-α levels, very similar to healthy controls (48). These observations are similar to our results in which we could not observe the individual variations in healthy donors’ DCs infected with *L. infantum*. Another possibility is that even with similar rates of infection and parasites per cell, our results reflect the differences cited before concerning the immunogenicity of *L. braziliensis* and *L. infantum*.

We observed that *L. infantum* caused an inhibition of DC apoptosis. The same phenomenon was not observed for *L. braziliensis*. This demonstrates that possibly only *L. infantum* was able to stimulate the development of an environment adequate for the multiplication inside the cells and that favors a possible spread to other organs as bone marrow and liver. This surviving of DC was also seen in *L. mexicana* infection when apoptosis was induced by camptothecin (49). Indeed, DCs infected with *L. mexicana* amastigotes decreased the phosphorylation of MAP kinase p38 and JNK, causing a decreased DNA fragmentation in the camptothecin stimulated DCs. *L. mexicana* amastigotes activated antiapoptotic pathways, such as PI3K and AKT, allowing the inhibition of infected DCs’ cell death (50). The opposite effect was observed with the bacteria *Brucella abortus* that induced apoptosis and necrosis of murine DCs. This apoptosis regulation was performed by caspase-2 in mouse model (51).

A very elegant paper showed that Dectin-1, MR, and DC-SIGN homolog SIGNR3 are able to recognize *L. infantum*, but only DC-SIGN (CD209) and MR triggered pro-IL1 beta processing in a caspase-1-dependent way, being crucial for microbicidal activity of macrophages. On the other hand, signaling through SIGNR3 seems to favor parasite survival because of its modulation of inflammasome activation (44). Once we found a possible correlation of CD209 and cell death, perhaps the internalization of *L. infantum* by CD209 in human DCs could also be interfering in the activation of caspase-1, causing the survival of DCs differently from the data observed on macrophages. On the other hand, *L. braziliensis* seems to not interfere in this pathway of cell death regulation, once we did not observe differences concerning DC survival.

In this way, we can conclude that both *L. infantum* and *L. braziliensis* possibly enter DCs through DC-SIGN, causing a positive regulation on CD86 expression as well as on TNF-α production after this initial interaction. Nevertheless, only *L. infantum* induced a survival in human DCs, probably favoring its establishment and spread in the host, differently from *L. braziliensis* that seems to be more silent in these initial events concerning DC survival. This work shed light in the differences between these two parasites and their interactions with human DCs and opens the field to study the development of intracellular signaling pathways responsible for these phenomena.

## Author Contributions

SF and CF: conceived and designed the experiments. SF, TJ, LF, DB, and CF: performed the experiments. SF, TJ, and CF: analyzed the data. SF, JS, and CF: contributed reagents/materials/analysis tools. SF and CF: wrote the paper.

## Conflict of Interest Statement

The authors declare that the research was conducted in the absence of any commercial or financial relationships that could be construed as a potential conflict of interest.

## References

[1] WHO. Accelerating Work to Overcome the Global Impact of Neglected Tropical. Geneva: WHO (2012).

[2] Alvar J Vélez D Bern C Herrero M Desjeux P Cano J Leishmaniasis worldwide and global estimates of its incidence. PLoS One (2012) 7:e35671. 10.1371/journal.pone.0035671 22693548 PMC3365071

[3] Santos VC Vale VF Silva SM Nascimento AA Saab NA Soares RP Host modulation by a parasite: how *Leishmania infantum* modifies the intestinal environment of *Lutzomyia longipalpis* to favor its development. PLoS One (2014) 9:e111241. 10.1371/journal.pone.0111241 25365351 PMC4218848

[4] Ramos PK Brito MV Silveira FT Salgado CG De Souza W Picanço-Diniz CW In vitro cytokines profile and ultrastructural changes of microglia and macrophages following interaction with *Leishmania*. Parasitology (2014) 141:1052–63. 10.1017/S0031182014000274 24717447

[5] Argueta-Donohué J Wilkins-Rodríguez AA Aguirre-García M Gutiérrez-Kobeh L Differential phagocytosis of *Leishmania mexicana* promastigotes and amastigotes by monocyte-derived dendritic cells. Microbiol Immunol (2015). 60:369–81. 10.1111/1348-0421.12325 26399218

[6] Prina E Abdi S Lebastard M Perret E Winter N Antoine J. Dendritic cells as host cells for the promastigote and amastigote stages of *Leishmania amazonensis*: the role of opsonins in parasite uptake and dendritic cell maturation. J Cell Sci (2004) 117:315–25. 10.1242/jcs.00860 14657281

[7] Barral A Barral-Netto M Almeida R de Jesus AR Grimaldi Júnior G Netto EM Lymphadenopathy associated with *Leishmania braziliensis* cutaneous infection. Am J Trop Med Hyg (1992) 47:587–92. 1449199 10.4269/ajtmh.1992.47.587

[8] Carvalho AM Amorim CF Barbosa JL Lago AS Carvalho EM. Age modifies the immunologic response and clinical presentation of American tegumentary leishmaniasis. Am J Trop Med Hyg (2015) 92:1173–7. 10.4269/ajtmh.14-0631 25918209 PMC4458822

[9] Ready PD. Epidemiology of visceral leishmaniasis. Clin Epidemiol (2014) 6:147–54. 10.2147/CLEP.S44267 24833919 PMC4014360

[10] Bhattacharya SK Dash AP Treatment of visceral leishmaniasis: options and choice. Lancet Infect Dis (2016) 16:142–3. 10.1016/S1473-3099(15)00528-9 26867454

[11] Gollob KJ Viana AG Dutra WO. Immunoregulation in human American leishmaniasis: balancing pathology and protection. Parasite Immunol (2014) 36:367–76. 10.1111/pim.12100 24471648 PMC4113557

[12] Cools N Ponsaerts P Van Tendeloo VF Berneman ZN. Balancing between immunity and tolerance: an interplay between dendritic cells, regulatory T cells, and effector T cells. J Leukoc Biol (2007) 82:1365–74. 10.1189/jlb.0307166 17711977

[13] Colmenares M Puig-Kröger A Pello OM Corbí AL Rivas L. Dendritic cell (DC)-specific intercellular adhesion molecule 3 (ICAM-3)-grabbing nonintegrin (DC-SIGN, CD209), a C-type surface lectin in human DCs, is a receptor for *Leishmania amastigotes*. J Biol Chem (2002) 277:36766–9. 10.1074/jbc.M205270200 12122001

[14] Vannberg FO Chapman SJ Khor CC Tosh K Floyd S Jackson-Sillah D CD209 genetic polymorphism and tuberculosis disease. PLoS One (2008) 3:e1388. 10.1371/journal.pone.0001388 18167547 PMC2148105

[15] Jin W Li C Du T Hu K Huang X Hu Q. DC-SIGN plays a stronger role than DCIR in mediating HIV-1 capture and transfer. Virology (2014) 458-459:83–92. 10.1016/j.virol.2014.04.016 24928041

[16] Liese J Schleicher U Bogdan C TLR9 signaling is essential for the innate NK cell response in murine cutaneous leishmaniasis. Eur J Immunol (2007) 37:3424–34. 10.1002/eji.200737182 18034422

[17] Dalod M Chelbi R Malissen B Lawrence T. Dendritic cell maturation: functional specialization through signaling specificity and transcriptional programming. EMBO J (2014) 33:1104–16. 10.1002/embj.201488027 24737868 PMC4193918

[18] Steinman RM Turley S Mellman I Inaba K The induction of tolerance by dendritic cells that have captured apoptotic cells. J Exp Med (2000) 191:411–6. 10.1084/jem.191.3.411 10662786 PMC2195815

[19] Vargas-Inchaustegui DA Xin L Soong L. *Leishmania braziliensis* infection induces dendritic cell activation, ISG15 transcription, and the generation of protective immune responses. J Immunol (2008) 180:7537–45. 10.4049/jimmunol.180.11.7537 18490754 PMC2641013

[20] Neves BM Silvestre R Resende M Ouaissi A Cunha J Tavares J Activation of phosphatidylinositol 3-kinase/Akt and impairment of nuclear factor-kappaB: molecular mechanisms behind the arrested maturation/activation state of *Leishmania infantum*-infected dendritic cells. Am J Pathol (2010) 177:2898–911. 10.2353/ajpath.2010.100367 21037075 PMC2993270

[21] Resende M Moreira D Augusto J Cunha J Neves B Cruz MT *Leishmania*-infected MHC class II high dendritic cells polarize CD4+ T cells toward a nonprotective T-bet+ IFN-γ+ IL-10+ phenotype. J Immunol (2013) 191:262–73. 10.4049/jimmunol.1203518 23729437

[22] Marovich MA McDowell MA Thomas EK Nutman TB. IL-12p70 production by *Leishmania major*-harboring human dendritic cells is a CD40/CD40 ligand-dependent process. J Immunol (2000) 164:5858–65. 10.4049/jimmunol.164.11.5858 10820265

[23] Brown JA Greenwald RJ Scott S Schweitzer AN Satoskar AR Chung C T helper differentiation in resistant and susceptible B7-deficient mice infected with *Leishmania major*. Eur J Immunol (2002) 32:1764–72. 10.1002/1521-4141(200206)32:6<1764::AID-IMMU1764>3.0.CO;2-V 12115660

[24] Carvalho AK Carvalho K Passero LF Sousa MG da Matta VL Gomes CM Differential recruitment of dendritic cells subsets to lymph nodes correlates with a protective or permissive T-cell response during *Leishmania* (*Viannia*) *braziliensis* or *Leishmania* (*Leishmania*) *amazonensis* infection. Mediators Inflamm (2016) 2016:7068287. 10.1155/2016/7068287 27073297 PMC4814687

[25] Favali C Tavares N Clarêncio J Barral A Barral-Netto M Brodskyn C. *Leishmania amazonensis* infection impairs differentiation and function of human dendritic cells. J Leukoc Biol (2007) 82:1401–6. 10.1189/jlb.0307187 17890507

[26] Soong L. Modulation of dendritic cell function by *Leishmania* parasites. J Immunol (2008) 180:4355–60. 10.4049/jimmunol.180.7.4355 18354154 PMC2639709

[27] Ishii N Ndhlovu LC Murata K Sato T Kamanaka M Sugamura K OX40 (CD134) and OX40 ligand interaction plays an adjuvant role during in vivo Th2 responses. Eur J Immunol (2003) 33:2372–81. 10.1002/eji.200324031 12938213

[28] Akiba H Miyahira Y Atsuta M Takeda K Nohara C Futagawa T Critical contribution of OX40 ligand to T helper cell type 2 differentiation in experimental leishmaniasis. J Exp Med (2000) 191:375–80. 10.1084/jem.191.2.375 10637281 PMC2195752

[29] Tuladhar R Oghumu S Dong R Peterson A Sharpe AH Satoskar AR. Ox40L-Ox40 pathway plays distinct roles in regulating Th2 responses but does not determine outcome of cutaneous leishmaniasis caused by *Leishmania mexicana* and *Leishmania major*. Exp Parasitol (2015) 148:49–55. 10.1016/j.exppara.2014.11.002 25447125 PMC4474147

[30] Liang SC Greenwald RJ Latchman YE Rosas L Satoskar A Freeman GJ PD-L1 and PD-L2 have distinct roles in regulating host immunity to cutaneous leishmaniasis. Eur J Immunol (2006) 36:58–64. 10.1002/eji.200535458 16358363

[31] Winau F Weber S Sad S de Diego J Hoops SL Breiden B Apoptotic vesicles crossprime CD8 T cells and protect against tuberculosis. Immunity (2006) 24:105–17. 10.1016/j.immuni.2005.12.001 16413927

[32] Gutiérrez-Kobeh L de Oyarzabal E Argueta J Wilkins A Salaiza N Fernández E Inhibition of dendritic cell apoptosis by *Leishmania mexicana* amastigotes. Parasitol Res (2013) 112:1755–62. 10.1007/s00436-013-3334-2 23420408

[33] Bieber K Autenrieth SE. Insights how monocytes and dendritic cells contribute and regulate immune defense against microbial pathogens. Immunobiology (2015) 220:215–26. 10.1016/j.imbio.2014.10.025 25468558

[34] Xin L Li K Soong L. Down-regulation of dendritic cell signaling pathways by *Leishmania amazonensis* amastigotes. Mol Immunol (2008) 45:3371–82. 10.1016/j.molimm.2008.04.018 18538399 PMC2583126

[35] Contreras I Estrada JA Guak H Martel C Borjian A Ralph B Impact of *Leishmania mexicana* infection on dendritic cell signaling and functions. PLoS Negl Trop Dis (2014) 8:e3202. 10.1371/journal.pntd.0003202 25255446 PMC4177750

[36] Brodskyn C Beverley SM Titus RG. Virulent or avirulent (dhfr-ts-) *Leishmania major* elicit predominantly a type-1 cytokine response by human cells in vitro. Clin Exp Immunol (2000) 119:299–304. 10.1046/j.1365-2249.2000.01122.x 10632666 PMC1905512

[37] Roy S Mukhopadhyay D Mukherjee S Ghosh S Kumar S Sarkar K A defective oxidative burst and impaired antigen presentation are hallmarks of human visceral leishmaniasis. J Clin Immunol (2015) 35:56–67. 10.1007/s10875-014-0115-3 25479930

[38] Vieira ÉL Keesen TS Machado PR Guimarães LH Carvalho EM Dutra WO Immunoregulatory profile of monocytes from cutaneous leishmaniasis patients and association with lesion size. Parasite Immunol (2013) 35:65–72. 10.1111/pim.12012 23050581 PMC3575026

[39] Revest M Donaghy L Cabillic F Guiguen C Gangneux JP. Comparison of the immunomodulatory effects of *L. donovani* and *L. major* excreted-secreted antigens, particulate and soluble extracts and viable parasites on human dendritic cells. Vaccine (2008) 26:6119–23. 10.1016/j.vaccine.2008.09.005 18804505

[40] Colmenares M Corbí AL Turco SJ Rivas L. The dendritic cell receptor DC-SIGN discriminates among species and life cycle forms of *Leishmania*. J Immunol (2004) 172:1186–90. 10.4049/jimmunol.172.2.1186 14707095

[41] Markikou-Ouni W Drini S Bahi-Jaber N Chenik M Meddeb-Garnaoui A. Immunomodulatory effects of four *Leishmania infantum* potentially excreted/secreted proteins on human dendritic cells differentiation and maturation. PLoS One (2015) 10:e0143063. 10.1371/journal.pone.0143063 26581100 PMC4651425

[42] Green PJ Feizi T Stoll MS Thiel S Prescott A McConville MJ. Recognition of the major cell surface glycoconjugates of *Leishmania* parasites by the human serum mannan-binding protein. Mol Biochem Parasitol (1994) 66:319–28. 10.1016/0166-6851(94)90158-9 7808481

[43] Liu D Uzonna JE. The early interaction of *Leishmania* with macrophages and dendritic cells and its influence on the host immune response. Front Cell Infect Microbiol (2012) 2:83. 10.3389/fcimb.2012.00083 22919674 PMC3417671

[44] Lefèvre L Lugo-Villarino G Meunier E Valentin A Olagnier D Authier H The C-type lectin receptors dectin-1, MR, and SIGNR3 contribute both positively and negatively to the macrophage response to *Leishmania infantum*. Immunity (2013) 38:1038–49. 10.1016/j.immuni.2013.04.010 23684988

[45] Carvalho K Vallejo M Camargo Z Puccia R. Use of recombinant gp43 isoforms expressed in *Pichia pastoris* for diagnosis of paracoccidioidomycosis. Clin Vaccine Immunol (2008) 15:622–9. 10.1128/CVI.00437-07 18235042 PMC2292662

[46] de Assis Souza M de Castro MC de Oliveira AP de Almeida AF de Almeida TM Reis LC Cytokines and NO in American tegumentary leishmaniasis patients: profiles in active disease, after therapy and in self-healed individuals. Microb Pathog (2013) 57:27–32. 10.1016/j.micpath.2013.02.004 23428929

[47] Oliveira WN Ribeiro LE Schrieffer A Machado P Carvalho EM Bacellar O. The role of inflammatory and anti-inflammatory cytokines in the pathogenesis of human tegumentary leishmaniasis. Cytokine (2014) 66:127–32. 10.1016/j.cyto.2013.12.016 24485388 PMC4047562

[48] Nateghi Rostami M Seyyedan Jasbi E Khamesipour A Mohammadi AM Tumour necrosis factor-alpha (TNF-α) and its soluble receptor type 1 (sTNFR I) in human active and healed leishmaniases. Parasite Immunol (2016) 38:255–60. 10.1111/pim.12305 26813918

[49] Valdés-Reyes L Argueta J Morán J Salaiza N Hernández J Berzunza M *Leishmania mexicana*: inhibition of camptothecin-induced apoptosis of monocyte-derived dendritic cells. Exp Parasitol (2009) 121:199–207. 10.1016/j.exppara.2008.10.020 19041644

[50] Vázquez-López R Argueta-Donohué J Wilkins-Rodríguez A Escalona-Montaño A Aguirre-García M Gutiérrez-Kobeh L. *Leishmania mexicana* amastigotes inhibit p38 and JNK and activate PI3K/AKT: role in the inhibition of apoptosis of dendritic cells. Parasite Immunol (2015) 37:579–89. 10.1111/pim.12275 26352010

[51] Li X He Y. Caspase-2-dependent dendritic cell death, maturation, and priming of T cells in response to *Brucella abortus* infection. PLoS One (2012) 7:e43512. 10.1371/journal.pone.0043512 22927979 PMC3425542

